# The Importance of Aquaporin 1 in Pancreatitis and Its Relation to the CFTR Cl^-^ Channel

**DOI:** 10.3389/fphys.2018.00854

**Published:** 2018-07-12

**Authors:** Viktória Venglovecz, Petra Pallagi, Lajos V. Kemény, Anita Balázs, Zsolt Balla, Eszter Becskeházi, Eleonóra Gál, Emese Tóth, Ágnes Zvara, László G. Puskás, Katalin Borka, Matthias Sendler, Markus M. Lerch, Julia Mayerle, Jens-Peter Kühn, Zoltán Rakonczay, Péter Hegyi

**Affiliations:** ^1^Department of Pharmacology and Pharmacotherapy, University of Szeged, Szeged, Hungary; ^2^First Department of Medicine, University of Szeged, Szeged, Hungary; ^3^Department of Pathophysiology, University of Szeged, Szeged, Hungary; ^4^Laboratory of Functional Genomics, Biological Research Centre, Hungarian Academy of Sciences, Szeged, Hungary; ^5^Second Department of Pathology, Semmelweis University, Budapest, Hungary; ^6^Department of Medicine A, University Medicine Greifswald, University of Greifswald, Greifswald, Germany; ^7^Department of Medicine II, Klinikum Grosshadern, Universitätsklinikum der Ludwig-Maximilians-Universität München, Munich, Germany; ^8^Institute of Radiology, University Medicine Greifswald, University of Greifswald, Greifswald, Germany; ^9^Institute and Policlinic of Radiology, University Hospital Carl Gustav Carus, TU Dresden, Dresden, Germany; ^10^MTA-SZTE Translational Gastroenterology Research Group, University of Szeged, Szeged, Hungary; ^11^Institute for Translational Medicine and First Department of Medicine, Medical School, University of Pécs, Pécs, Hungary

**Keywords:** pancreas, aquaporins, bile acids, HCO_3_^-^ secretion, CFTR Cl^-^ channel

## Abstract

Aquaporins (AQPs) facilitate the transepithelial water flow involved in epithelial fluid secretion in numerous tissues; however, their function in the pancreas is less characterized. Acute pancreatitis (AP) is a serious disorder in which specific treatment is still not possible. Accumulating evidence indicate that decreased pancreatic ductal fluid secretion plays an essential role in AP; therefore, the aim of this study was to investigate the physiological and pathophysiological role of AQPs in the pancreas. Expression and localization of AQPs were investigated by real-time PCR and immunocytochemistry, whereas osmotic transmembrane water permeability was estimated by the dye dilution technique, in Capan-1 cells. The presence of AQP1 and CFTR in the mice and human pancreas were investigated by immunohistochemistry. Pancreatic ductal HCO_3_^-^ and fluid secretion were studied on pancreatic ducts isolated from wild-type (WT) and AQP1 knock out (KO) mice using microfluorometry and videomicroscopy, respectively. *In vivo* pancreatic fluid secretion was estimated by magnetic resonance imaging. AP was induced by intraperitoneal injection of cerulein and disease severity was assessed by measuring biochemical and histological parameters. In the mice, the presence of AQP1 was detected throughout the whole plasma membrane of the ductal cells and its expression highly depends on the presence of CFTR Cl^-^ channel. In contrast, the expression of AQP1 is mainly localized to the apical membrane of ductal cells in the human pancreas. Bile acid treatment dose- and time-dependently decreased mRNA and protein expression of AQP1 and reduced expression of this channel was also demonstrated in patients suffering from acute and chronic pancreatitis. HCO_3_^-^ and fluid secretion significantly decreased in AQP1 KO versus WT mice and the absence of AQP1 also worsened the severity of pancreatitis. Our results suggest that AQP1 plays an essential role in pancreatic ductal fluid and HCO_3_^-^ secretion and decreased expression of the channel alters fluid secretion which probably contribute to increased susceptibility of the pancreas to inflammation.

## Introduction

The pancreas secretes 1.5–2 l of fluid per day (Hegyi et al., 2011a; Pallagi et al., 2015). This fluid contains the inactive form of digestive enzymes and is prominently rich in HCO_3_^-^ produced by the ductal cells (Pallagi et al., 2015). The main functions of this HCO_3_^-^-rich fluid are (i) to prevent the premature activation of zymogens (Pallagi et al., 2011), (ii) to wash out the toxic factors (such as bile acids) from the ductal tree (Venglovecz et al., 2008), and (iii) to provide an alkaline environment in the duodenum for the optimal function of digestive enzymes (Steward et al., 2005). In the last few years, the importance of ion transport proteins has been highlighted in the course of pancreatitis; therefore intensive research has been conducted in order to characterize their pathological roles (Hegyi and Rakonczay, 2015; Pallagi et al., 2015). These clinical and experimental studies indicate that impaired ductal HCO_3_^-^ secretion makes the pancreas more susceptible to inflammatory diseases such as acute or chronic pancreatitis (CP) (Hegyi and Rakonczay, 2010; Hegyi et al., 2011b; Takacs et al., 2013; Pallagi et al., 2014; Maleth et al., 2015). In contrast, much less is known about the water transport processes, despite the fact that movement of electrolytes is osmotically coupled to water flow.

Aquaporins (AQPs) are small membrane proteins that primarily mediate the transport of water molecules and recent research also emphasize their importance in certain regulatory processes (Rodriguez et al., 2011; Ribatti et al., 2014; Madeira et al., 2015). In mammals, 13 AQP isoforms have been identified so far and a few of them show a species-specific expression pattern in the pancreas. In humans, AQP1, -5, -8, and -12 are present in the pancreas (Hurley et al., 2001; Tani et al., 2001; Furuya et al., 2002; Burghardt et al., 2003; Itoh et al., 2005). AQP1 is the first AQP which has been described and is exclusively permeable to water (Preston et al., 1992). The presence of this channel has been shown in the apical and lateral plasma membrane of centroacinar cells, and in the apical and basolateral membranes of intercalated and intralobular ducts (Furuya et al., 2002; Burghardt et al., 2003). AQP5 is an aquaglyceroporin which mediates the transport of glycerol, urea and other small solutes beside water. Expression of AQP5 has only been detected in the apical plasma membrane of intercalated ducts but not in the centroacinar cells (Burghardt et al., 2003). AQP1 and -5 are colocalized with the cystic fibrosis transmembrane conductance regulator (CFTR) Cl^-^ channel at the apical membrane of the ductal cells which indicates that these channels influence each other’s function (Burghardt et al., 2003). AQP8 and -12 are exclusively localized to centroacinar cells. AQP8 is expressed on the apical plasma membrane (Hurley et al., 2001; Tani et al., 2001), whereas AQP12, a relatively new member of the AQP family, is an intracellular water channel (Itoh et al., 2005). Several studies suggest that altered expression or functions of AQPs are often associated with different diseases affecting the kidney, colon, lacrimal, or salivary glands (Steinfeld et al., 2001; Tsubota et al., 2001; Bedford et al., 2003; Zhu et al., 2016). However, only scarce information is available regarding the role of AQPs in pancreatitis. Ohta et al. (2009) have shown that in the absence of AQP12 the course of cerulein-induced pancreatitis is much worse, probably due to the defect in the secretion of zymogens. In addition, decreased expression of AQP1 and -8 has been found in this pancreatitis model that may also affects the outcome of pancreatitis (Kitami et al., 2007).

The aim of this study is to provide the first detailed characterization regarding the role of AQPs in the pancreas, both under physiological and pathophysiological conditions and to study its relationship to the CFTR Cl^-^ channel. We have chosen to focus on AQP1, since this isoform is abundantly expressed in both acinar and ductal cells of mouse and human pancreas and the role of this AQP has not been evaluated in pancreatitis yet. Using *in vitro* models, human tissues and transgenic mice, we have shown that AQP1 plays essential role in ductal fluid and HCO_3_^-^ secretion, lack of CFTR decreases its expression and deletion of AQP1 is strongly associated with increased susceptibility of the gland to pancreatitis.

## Materials and Methods

### Ethical Approval

Animal experiments were conducted in accordance with the Guide for the Care and Use of Laboratory Animals (United States, Department of Health and Human Services). In addition, the experimental protocol was approved by the local Ethical Board of the University of Szeged, Hungary and by the National Scientific Ethical Committee on Animal Experimentation (Budapest, Hungary). The use of human tissue was approved by the local ethical committee (University of Szeged, Hungary) and written informed consent was obtained from the patients.

### Transgenic Mice

AQP1 knock out (KO) mice were a kind gift from Dr. Alan Verkman (University of California, CA, United States) and Dr. Alastair Poole (University of Bristol, United Kingdom). CFTR KO mice were kindly supplied by Dr. Ursula Seidler (Hannover Medical School, Hannover, Germany). Animals were kept in standard plastic cages on 12:12 h light-dark cycle at room temperature (23 ± 1°C) and had free access to standard or CFTR specific laboratory chow and drinking solutions. Functional experiments were performed on litter-matched (age 12–16 weeks, both sexes) wild-type (WT) and AQP1 KO mice. All mice were genotyped prior to the experiments. For genotyping, genomic DNA from the tail was isolated and amplified by traditional PCR.

### Human Pancreatic Tissue Samples

Human pancreatic tissue samples were obtained from autopsy and from surgical resections. Control tissue (*n* = 5) were collected from the tumor-free region of the pancreas of patients with neuroendocrine tumors. Tissue samples from patients with acute necrotizing pancreatitis (ANP; *n* = 5) or CP (*n* = 5) were from autopsy and from surgical resections. The average age of ANP patients was 56 ± 2.8 years, and the male/female ratio was 1.5:1. The average age of CP patients was 56.8 ± 2.8 years, and the male/female ratio was 4:1.

### Cell Cultures and Treatments

Capan-1, Panc-1, and Miapaca-2 cells were obtained from the American Type Culture Collection (Manassas, VA, United States). Capan-1 cells were maintained in Roswell Park Memorial Institute (RPMI)-1640 Medium supplemented with 15% (v/v) fetal bovine serum (FBS), 1% (v/v) L-glutamine and 1% (v/v) Penicillin–Streptomycin (PS). Panc-1 and Miapaca-2 were maintained in Dulbecco’s Modified Eagle’s Medium (DMEM) high glucose Medium supplemented with 10% (v/v) FBS, 1% (v/v) L-glutamine, 2.5% (v/v) horse serum and 1% (v/v) PS. All three cell lines were kept in a humidified incubator at 37°C. Cells from passage numbers 20–60 were used in this study. In case of PCR experiments 10^6^, whereas in the case of immunostaining 10^4^ cells were seeded into 75 cm^2^ tissue culture flasks or glass bottom petri dishes, respectively, and incubated for 24 h at 37°C. After the incubation, cells were treated with chenodeoxycholic acid (CDCA; 100, 300, and 500 μM), glycochenodeoxycholic acid (GCDCA; 100, 300, and 500 μM), ethanol (EtOH; 1, 10, and 100 mM), palmitoleic acid (POA; 10, 100, and 200 μM) and palmitoleic acid ethyl ester (POAEE; 10, 100 and 200 μM) for 6, 12, 24, and 48 h and the mRNA and protein expression were investigated by real-time PCR and immunohistochemistry.

### Chemicals and Solutions

2,7-bis-(2-carboxyethyl)-5(6)-carboxyfluorescein acetoxymethyl ester (BCECF-AM) was from Invitrogen (Eugene, OR, United States). BCECF-AM (2 mmol/l) were prepared in dimethyl sulfoxide (DMSO) and stored at -20°C. POAEE was purchased from Cayman Chemical (Tallinn, Estonia). POA and POAEE were made up as a 10 mM stock solution in DMSO and stored at -20°C. Chromatographically pure collagenase was purchased from Worthington (Lakewood, NJ, United States). All other chemicals were obtained from Sigma-Aldrich (Budapest, Hungary).

For microfluorimetry studies, the standard HEPES-buffered solution contained (in mM): 130 NaCl, 5 KCl, 1 CaCl_2_, 1 MgCl_2_, 10 D-glucose and 10 Na-HEPES. HEPES-buffered solutions were gassed with 100% O_2_ and their pH was set to 7.4 with HCl. The standard HCO_3_^-^/CO_2_-buffered solution contained (in mM): 115 NaCl, 25 NaHCO_3_, 5 KCl, 1 CaCl_2_, 1 MgCl_2_, and 10 D-glucose. The Cl^-^-free HCO_3_^-^/CO_2_-buffered solution contained (in mM): 115 Na-gluconate, 25 NaHCO_3_, 6 Ca-gluconate, 1 Mg-gluconate, 2.5 K_2_H-sulfate, and 10 D-glucose. HCO_3_^-^/CO_2_-buffered solutions were gassed with 95% O_2_/5% CO_2_ to set the pH to 7.4.

### Isolation of Pancreatic Ducts and Measurement of Intracellular pH

Intra/interlobular ducts were isolated from the pancreas of WT and AQP KO mice using the microdissection technique as described previously (Argent et al., 1986). Changes in intracellular pH (pH_i_) were detected using the pH-sensitive fluorescence dye, BCECF. Pancreatic ducts were incubated with BCECF-AM (2 μM) for 30–60 min, at room temperature. After the incubation, ducts were attached to a cover glass, which formed the base of a perfusion chamber, mounted on the stage of an IX71 live cell imaging fluorescence microscope (Olympus, Budapest, Hungary) and excited at 440 and 490 nm. Emissions were monitored at 530 nm. Five to seven region of interests (ROIs) were examined in each experiment, and one measurement per second was obtained. The 490/440 fluorescence ratio was calibrated to pH_i_ using the high K^+^-nigericin technique, as previously described (Thomas et al., 1979; Hegyi et al., 2004).

### Measurement of HCO_3_^-^ Secretion

In order to estimate HCO_3_^-^ efflux, the activity of the Cl^-^/HCO_3_^-^ exchanger was measured by the Cl^-^ withdrawal technique. Removal of luminal Cl^-^ from the standard HCO_3_^-^/CO_2_-buffered solution induced an alkalization in the cells due to the reverse mode of the exchanger. Re-addition of Cl^-^ induces HCO_3_^-^ secretion via the Cl^-^/HCO_3_^-^ exchanger. Under these conditions the initial rate of acidification reflects the activity of the Cl^-^/HCO_3_^-^ exchanger. In order to evaluate base efflux [-*J*(B^-^/min)] the following equation was used: -*J*(B^-^/min) = ΔpH/Δt × β_total_, where ΔpH/Δt is the rate of acidification measured over the first 60 s and β_total_ is the total buffering capacity of the cell.

### Measurement of *in Vitro* Ductal Fluid Secretion

Fluid secretion of intra/interlobular pancreatic ducts was measured using a swelling method, as described previously (Fernandez-Salazar et al., 2004). Briefly, isolated pancreatic ducts were attached to a cover glass which formed the base of a perfusion chamber and mounted on the stage of an IX71 live cell imaging fluorescence microscope. Low magnification, bright-field images were acquired at 1-min intervals using a CCD camera (Hamamatsu ORCA-ER, Olympus, Budapest, Hungary). At the end of each experiment, ducts were perfused with hypotonic solution in order to check the integrity of the duct wall. Ducts that not respond to the hypotonic challenge were excluded from the analysis. Changes in relative luminal volume was analyzed by Scion Image software (Scion Corporation, Frederick, MD, United States) (Fernandez-Salazar et al., 2004; Pascua et al., 2009).

### Measurement of *in Vivo* Pancreatic Fluid Secretion

In order to measure pancreatic fluid secretion *in vivo*, magnetic resonance imaging (MRI) was performed on WT and AQP1 KO mice (Maleth et al., 2015). Animals were allowed free access to pineapple juice 12 h before the MRI examination. MRI was performed in a 7.1 Tesla animal scanner (Bruker, Ettlingen, Germany). Strong T2-weighted series of the complete abdomen were acquired before and after retroorbital injection of 10 IU units/kg body weight (b.w.) secretin (ChiroStim, ChiRhoClin, Burtonville, MD, United States). The time between injection and MRI was 6 min. The sequences were acquired using the image parameters: TR/TE 4400/83 ms; flip angle: 180°; matrix 256 × 256; field of view 40 × 40 mm; bandwidth 315 Hz/pixel; slice thickness 1 mm; 20 slices. All image analyses were performed using Osirix (version 5; Pixameo, Bernex, Switzerland). In order to exclude effects of the basal secretion, MRI datasets after and before secretion were subtracted. The created images show the total excretion after secretin stimulation. Excreted fluid is defined as high signal intensity in created images. The fluid excretion into the small intestine was segmented in each slice. The software calculated the volume of the segmented areas. This volume represents the total excreted volume (TEV). In order to minimize artifacts, image noise was reduced.

### Measurement of Osmotic Transepithelial Water Permeability

The osmotic transcellular water movement (P_f_) was estimated using the cell-impermeant dye, Texas Red^TM^ Dextran as previously described (Levin et al., 2006). Briefly, cells (5 × 10^5^) were grown on a polyester permeable support (Transwell, 12 mm diameter and 0.4 μm pore size). Monolayer confluence was checked by measuring the transepithelial electrical resistance (R_T_) using an EVOM-G Volt/Ohm Meter (World Precision Instruments, Sarasota, FL, United States). Cells were washed with isoosmolar phosphate-buffered saline (PBS) from the basolateral surface and hyperosmolar PBS (complemented with 300 mM D-mannitol and 0.25 μg/mL Texas Red Dextran) from the luminal surface. In some experiments, different concentrations of bile acids were added to the apical solution. Transwells were than placed into a CO_2_ incubator and 5 μl samples were collected from the apical solution at specified time points. Water moves along the osmotic gradient that causes the dilution of the fluorescent dye, in the apical solution. Fluorescence was measured at 595 nm excitation and 615 nm emission, using a Fluoro Max-4 spectrofluorometer (Horiba Scientific, Tokyo, Japan). For the calculation of P_f_, the following formula was used: dV(0)/dt = P_f_^∗^S^∗^v_w_^∗^(Φ_1_-Φ_2_), where S is the tissue surface area, v_w_ is the partial molar volume of water and Φ_1_-Φ_2_ is the transepithelial osmotic gradient.

### Real-Time PCR

Total RNA was purified from individual cell culture samples using the RNA isolation kit of Macherey-Nagel (Macherey-Nagel, Düren, Germany). All the preparation steps were carried out according the manufacturer’s instructions. RNA samples were stored at –80°C in the presence of 30 U of Prime RNAse inhibitor (Fermentas, Lithuania) for further analysis. The quantity of isolated RNA samples was checked by spectrophotometry (NanoDrop 3.1.0, Rockland, DE, United States). In order to monitor gene expression, QRT-PCR was performed on a RotorGene 3000 instrument (Corbett Research, Sydney, NSW, Australia) using the TaqMan probe sets of specific Aquaporin genes (Applied Biosystems, Foster City, CA, United States). Information about the genes and the TaqMan assays is collected in **Table 1**. 3 μg of total RNA was reverse transcribed using the High-Capacity cDNA Archive Kit (Applied Biosystems, Foster City, CA, United States) according to the manufacturer’s instructions in final volume of 30 μL. The temperature profile of the reverse transcription was the following: 10 min at room temperature, 2 h at 37°C, 5 min on ice and finally 10 min at 75°C for enzyme inactivation. These steps were carried out in a Thermal Cycler machine (MJ Research, Waltham, MA, United States). After dilution with 30 μL of water, 1 μL of the diluted reaction mix was used as template in the QRT-PCR. For all the reactions TaqMan Universal Master Mix (Applied Biosystems, Foster City, CA, United States) were used according to the manufacturer’s instructions. Each reaction mixture (final volume 20 μL) contained 1 μL of primer-TaqMan probe mix. The QRT-PCR reactions were carried out under the following conditions: 15 min at 95°C and 45 cycles of 95°C for 15 s, 60°C for 1 min. Fluorescein dye (FAM) intensity was detected after each cycle. Non-template control sample was used for each PCR run to check the primer-dimer formation. Relative gene expression ratios were calculated as ΔCt values (Ct values of gene of interest versus Ct values of human hypoxanthine phosphoribosyltransferase gene). In the case of treatments, relative changes in gene expression were determined using the ΔΔC_T_ method as described in *Applied Biosystems User Bulletin* No. 2 (P/N 4303859). ΔΔC_T_ was calculated using the following formula: ΔΔC_T_= ΔC_T_ of treated cells – ΔC_T_ of control, non-treated cells. The N-fold differential expression in the target gene was expressed as 2^-ΔΔC_T_^. Genes with expression values less than or equal to 0.5 were considered to be down-regulated, whereas values higher than or equal to 2 were considered to be up-regulated. Values ranging from 0.51 to 1.99 were not considered to be significant.

**Table 1:** TaqMan assays used for the investigation of AQP expression.

Definition	Gene symbol	Ref Seq Acc. number	ABI TaqMan assay ID
Homo sapiens aquaporin 1 (Colton blood group)	AQP1	NM_198098	Hs00166067_m1
Homo sapiens aquaporin 2 (collecting duct)	AQP2	NM_000486	Hs00166640_m1
Homo sapiens aquaporin 3 (Gill blood group)	AQP3	NM_004925	Hs00185020_m1
Homo sapiens aquaporin 4	AQP4	NM_001650	Hs00242341_m1
Homo sapiens aquaporin 5	AQP5	NM_001651	Hs00387048_m1
Homo sapiens aquaporin 6, kidney specific	AQP6	NM_001652	Hs01546883_m1
Homo sapiens aquaporin 7	AQP7	NM_001170	Hs00357359_m1
Homo sapiens aquaporin 8	AQP8	NM_001169	Hs00154124_m1
Homo sapiens aquaporin 9	AQP9	NM_020980	Hs00175573_m1
Homo sapiens aquaporin 10	AQP10	NM_080429	Hs00369738_m1
Homo sapiens aquaporin 11	AQP11	NM_173039	Hs00542681_m1
Homo sapiens aquaporin 12A	AQP12A	NM_198998	Hs01651303_m1
Homo sapiens aquaporin 12B	AQP12B	NM_001102467.1	

### Immunocytochemistry

CAPAN-1 cells were washed with PBS twice and fixed in paraformaldehyde (4% in PBS) for 30 min at room temperature (RT). In order to avoid non-specific antibody binding cells were incubated with 10% donkey serum and 1% BSA for further 30 min. After blocking, cells were incubated with primary AQPs human, polyclonal antibodies (1:100 dilutions; Abcam, Cambridge, United Kingdom) at 4°C overnight. Petri dishes were then washed with PBS and incubated with FITC-conjugated AffiniPure Donkey Anti-Rabbit IgG secondary antibody (1:400 dilutions; DAKO, Milan, Italy) for 60 min at RT. Nuclei were counterstained with Dapi. Dishes were then mounted and observed by a Fluowiew 10i-W confocal microscopy (Olympus, Budapest, Hungary).

### Immunohistochemistry

Paraffin-embedded, 3- to 4-μm-thick sections of surgically removed resection specimens and autopsy tissue samples were used for immunohistochemistry. After deparaffinization of tissue samples with EZ Prep Concentrate 10X (Ventana Medical Systems, Tucson, AZ, United States), endogenous peroxidase blocking and antigen retrieval (CC1; Ventana Medical Systems), pancreas sections were incubated with polyclonal AQP1 antibody (1:100 dilution; Alomone Labs, Jerusalem, Israel) overnight at 4°C. Immunohistochemical staining was performed with horseradish peroxidase multimer-based, biotin-free detection technique according to the protocol of the automated Ventana system (Ventana Benchmark XT; Ventana Medical Systems). For visualization, the UltraView Universal diaminobenzidine (DAB) Detection Kit (Ventana Medical Systems) was applied. Sections from human pancreas were used as positive controls. For negative control, primary antibodies were substituted with antibody diluent (Ventana Medical Systems). The stained slides were digitized with Mirax Pannoramic MIDI and Mirax Pannoramic SCAN digital slide scanners (3DHistech Ltd., Budapest, Hungary).

Cryosections from WT, AQP1, and CFTR KO mice pancreas were fixed in 2% paraformaldehyde, permeabilized in 10% Tween 20-sodium citrate, blocked with 5% goat serum followed by immunofluorescent double staining for AQP1 mouse monoclonal antibody (1:500 dilutions; Thermo Fisher, Rockford, IL, United States) and CFTR rabbit polyclonal antibody (1:100 dilutions; Alomone Labs, Jerusalem, Israel) at 4°C, overnight. Following washing, sections were incubated with secondary antibodies goat-anti-mouse (Alexa fluor 488, Thermo Fisher, Rockford, IL, United States) and goat-anti-rabbit (Alexa fluor 568, Thermo Fisher, Rockford, IL, United States) for 2 h at room temperature in the dark. Nuclei were counterstained with Dapi. Sections were then mounted and analyzed using a Zeiss LSM 880 confocal laser scanning microscope (Carl Zeiss Technika Kft., Budaörs, Hungary).

### Quantification of the Immunostainings

In order to quantify AQP1 or CFTR positively stained area, 10–12 representative, digital images were taken from the human (normal pancreas, AP and CP) and mice (WT, AQP1 KO and CFTR KO) pancreas sections and from Capan-1 cells. Pictures were than converted to gray scale (16-bit) and thresholded in order to select the positively stained area, using the ImageJ software. The intensity of DAB (human pancreas) or fluorescence signal (mice pancreas and Capan-1 cells) varied on an arbitrary scale from 0 to 255, where 0 is the negative staining (white pixels) and 255 is the maximal staining (black pixels). In the human samples, the mean integrated density of the positively stained area was normalized to the mean integrated density of the total image and converted into percentage. In the cell line, the mean integrated density of the positively stained area was normalized to the total cell number and expressed in arbitrary units. In the mice samples, the ductal area was selected and the total fluorescence intensity was calculated and summarized which was than normalized to the ductal area (μm^2^) and expressed in arbitrary units/μm^2^. Comparison of each groups were calculated by one-way ANOVA, followed by the Holm-Sidak method. Statistical significance was defined as *p* ≤ 0.05. Data are expressed as means ± SEM.

### Induction of Acute Pancreatitis in Mice

Acute pancreatitis (AP) was induced in mice by hourly (10 times) intraperitoneal (i.p.) injections of cerulein (50 μg/kg) (Niederau et al., 1985; Ding et al., 2003; Pallagi et al., 2014) following anesthesia with i.p. 85 mg/bwkg pentobarbital (Bimeda MTC, Cambridge, ON, Canada). The control animals received the same amount of saline. Two hours after the final injection, mice were euthanized by pentobarbital overdose (200 mg/bwkg i.p.). Animals were exsanguinated through the cardiac puncture and the pancreata were immediately removed. The collected blood was centrifuged at 4°C with 2500 RCF for 15 min and the sera were stored at -20°C until use. The pancreas was trimmed from fat and lymphatic tissue, put into 6% neutral formaldehyde solution and stored at -80°C until use. Serum amylase activity was measured with a commercial colorimetric kit (Diagnosticum, Budapest, Hungary) with a FLUOstar OPTIMA (BMG Labtech, Budapest, Hungary) microplate reader at 405 nm. The formaldehyde-fixed pancreatic tissue was embedded in paraffin blocks, cut into 3 μm thick sections and stained for hematoxylin-eosin using standard techniques and viewed by light microscopy. A semiquantitative scoring system was used to evaluate the presence of edema, the rate of necrosis and infiltration of inflammatory cells according to the following scoring system (Kui et al., 2015): Edema (0: none, 1: patchy interlobular; 2: diffuse interlobular; 3: diffuse interlobular, and intraacinar), necrosis (0: none, 1: patchy interlobular; 2: diffuse interlobular; 3: diffuse interlobular and patchy intraacinar; 4: diffuse interlobular and intraacinar) and leukocytic infiltration (0: none; 1: patchy interlobular; 2: diffuse interlobular; 3: diffuse interlobular and intraacinar). The rate of necrosis was expressed as percentage of the total analyzed pancreatic area.

### Statistical Analysis

Data are expressed as means ± SEM. In the case of pancreatic fluid and HCO_3_^-^ secretion measurements, significant difference between groups was determined by ANOVA. Statistical analysis of the immunohistochemical data was performed using the Student’s *t*-test. Probability values of *p* ≤ 0.05 were accepted as being significant.

## Results

### Capan-1 Cells Express Functionally Active AQPs

**FIGURE 1:**
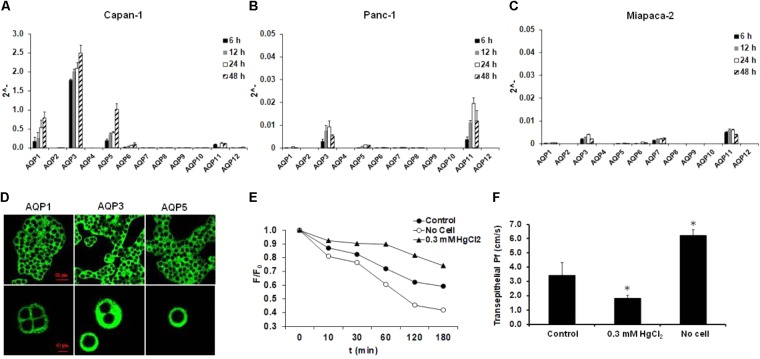
Expression and activity of AQPs in pancreatic ductal cells. Expression of different AQP isoforms was investigated by real-time PCR in **(A)** Capan-1, **(B)** Panc-1, and **(C)** Miapaca-2 pancreatic ductal cell lines, 6, 12, 24, and 48 h after the plating the cells. Data represent mean ± SEM of three, independent experiments. **(D)** Immunofluorescence staining of Capan-1 cells using FITC-conjugated anti-AQP1, -3, and -5 antibodies. **(E)** Osmotic water movement was investigated in Capan-1 cells. Representative graph shows changes in the fluorescence intensity of the apical solution, at different time points, in the present (triangle) and absence (black circle) of luminal HgCl_2_ (0.3 mM). To estimate the changes, the fluorescence intensity **(F)** was normalized to the initial value (F_0_). Transwell without cells was used for absolute positive control (open circle). **(F)** Summary of the P_f_ values obtained from the experiments in **(E)**. Data represent mean ± SEM of three, independent experiments. ^∗^*p* ≤ 0.05 vs. Control.

The relative gene expressions of AQP isoforms were studied in different pancreatic ductal cell lines (Capan-1, Panc-1, and Miapaca-2) using RT-PCR and TaqMan primer-probe sets, specific for AQP1-AQP12 isoforms (**Table 1**). Expressions of AQPs were investigated at different time points (6, 12, 24, and 48 h) 24 h after plating the cells, and as an internal gene, human hypoxanthine phosphoribosyltransferase (HPRT) was used. RT-PCR analysis revealed that among the three cell lines, AQPs were expressed at the highest level in Capan-1 cells, whereas in Panc-1 and Miapaca-2 the expression of AQPs was hardly detectable (**Figures 1A–C**). In Capan-1 cells high levels of AQP1, -3, and -5 was detected, which is in agreement with previous observations (Burghardt et al., 2003). The presence of these isoforms was also confirmed at protein level in the Capan-1 cells (**Figure 1D**). In order to test that the AQPs expressed on Capan-1 cells are functionally active, we investigated the transepithelial water flow in the presence and absence of the non-specific AQP inhibitor HgCl_2_. As shown in **Figures 1E,F**, administration of 0.3 mM HgCl_2_ to the apical solution inhibited the osmotic water movement by 52.6 ± 2.4%, indicating that significant proportion of water is transported through AQPs.

### AQP1 Is Essential for Pancreatic Ductal Fluid and HCO_3_^-^ Secretion

**FIGURE 2:**
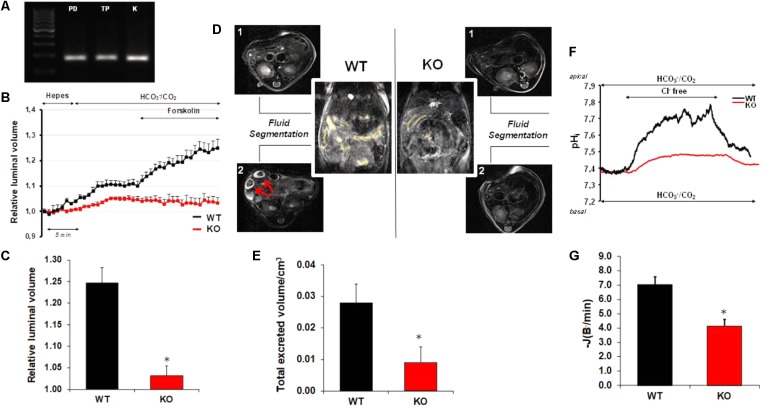
Pancreatic ductal HCO_3_^-^ and fluid secretion in AQP1 knock out mice. **(A)** Traditional PCR shows the presence of AQP1 mRNA both in the isolated pancreatic ducts (PD) and in the total pancreas (TP). As positive control, kidney was used (K). **(B)** Pancreatic ductal fluid secretion was measured on isolated pancreatic ducts from wild type (WT, black line) and AQP1 knock out (KO, red line) mice. Changes in the relative luminal volume of the ducts was measured by videomicroscopy and analyzed by Scion Image software. **(C)** Changes in the relative luminal volume of the pancreatic ducts of AQP1 WT and KO mice measured at the final time point (40 min). Data are shown as means ± SEM. *n* = 4–8 ducts/groups. ^∗^*p* ≤ 0.05 vs. WT. **(D)** Reconstructed images of the duodenal filling before (1) and after (2) secretin stimulation. Yellow color shows the presence of fluid. Red arrows indicate excreted volume in the small bowel after stimulation with secretin. In the case of KO mice, the secretin-stimulated fluid secretion significantly decreased. **(E)** *In vivo* pancreatic fluid secretion was measured as total excreted volume (TEV) using small animal magnetic resonance imaging. Raw data were acquired in axial and coronar view. Data are shown as means ± SEM. *n* = 4 animals/groups. ^∗^*p* ≤ 0.05 vs. WT. **(F)** Representative experimental traces show pancreatic ductal HCO_3_^-^ secretion in AQP1 WT (black line) and AQP1 KO (red line) mice. **(G)** Base flux [–*J*(B^-^/min)] was calculated from the ΔpH/Δt obtained by linear regression analysis of pH_i_ measurements made over the first 60 s after readdition of extracellular Cl^-^. Means ± SEM are from 20 ROIs of four ducts. ^∗^*p* ≤ 0.05 vs. WT.

Aquaporins are involved in transepithelial water transport in numerous tissues (Tradtrantip et al., 2009). In the next step, we investigated the role of this protein in pancreatic ductal fluid secretion using gene modified mice. Using traditional PCR we have shown the presence of AQP1 both in the isolated, intra/interlobular pancreatic ducts and the total pancreas (**Figure 2A**). Slight expression of AQP5 was also detected in the pancreatic ducts, whereas the mRNA presence of AQP8 and -12 was only observed in the total pancreas (data not shown). We decided to characterize the role of AQP1, since this isoform is constitutively expressed in both the ductal and acinar cells and also can be found in the mouse and human pancreas (**Table 2**). In contrast to AQP1, AQP3 is not expressed in the mouse pancreas and this isoform is either involved in tumor progression than pancreatitis (Direito et al., 2017; Huang et al., 2017). Although AQP5 is present in the intra/interlobular pancreatic ducts of mice and human, and probably plays role in ductal fluid secretion (Burghardt et al., 2003), KO animal for this isoform was not available to us. **Table 2** summarizes the presence of AQP isoforms in mice and human.

**Table 2:** mRNA expression of AQP isoforms in mice and human.

Known isoforms	Isoforms in mice pancreas	Isoforms in mice pancreatic ducts	Isoforms in human pancreas	Isoforms in human pancreatic ducts	Available KO mice
AQP0	–	–	–	–	–
AQP1	*√*	*√*	*√*	*√*	*√*
AQP2	–	–	–	–	–
AQP3	–	–	–	*√*	–
AQP4	–	–	–	–	*√*
AQP5	*√*	*√*	*√*	*√*	–
AQP6	–	–	–	–	–
AQP7	–	–	–	–	–
AQP8	*√*	–	*√*	–	–
AQP9	–	–	–	–	–
AQP10	–	–	–	–	–
AQP11	–	–	–	–	–
AQP12	*√*	–	*√*	–	–

The rate of fluid secretion was measured over 30–40 min using sealed ducts and the swelling technique. Initially, ducts were perfused with the standard HEPES-buffered solution and then perfusion was switched to HCO_3_^-^/CO_2_-buffered solution to initiate HCO_3_^-^-dependent secretion. As shown in **Figure 2B**, the presence of HCO_3_^-^ induced a dynamic swelling of the WT ducts as a result of fluid secretion into the closed luminal space. In contrast, ducts from AQP1 KO mice showed no or only a slight response to HCO_3_^-^. Under stimulated conditions (forskolin; 10 μM), the rate of fluid secretion further increased in the WT ducts, whereas in the case of KO ducts, no response was detected. **Figure 2C** summarizes the relative luminal volume changes in WT and KO ducts after forskolin stimulation. As shown in **Figure 2C**, the relative luminal volume increase reduced by 89.9 ± 6.2% in the absence of AQP1, indicating that this AQP isoform plays an essential role in fluid secretion.

We also investigated the rate of pancreatic fluid secretion *in vivo* by MRI cholangiopancreatography in anesthetized mice. Fluid secretion was stimulated by retroorbital administration of 10 U/kg b.w. secretin, and then TEV was measured in WT and KO animals. Similarly, to the *in vitro* measurements, the pancreatic fluid secretion significantly reduced in AQP1 KO (0.0041 TEV/cm^3^) vs. WT (0.023 TEV/cm^3^) mice (**Figures 2D,E**).

Next, we were curious whether the decreased ductal fluid secretion is also associated with impaired HCO_3_^-^ efflux in AQP1 KO animals. For the measurement of ductal HCO_3_^-^ secretion, intra/interlobular pancreatic ducts were isolated from the pancreas of WT and AQP1 KO mice. Ducts were perfused both from the luminal and basolateral membrane with HCO_3_^-^/CO_2_-buffered solution. Removal of Cl^-^ from the apical solution induced an alkalisation inside the cells, due to the reverse mode of the Cl^-^/HCO_3_^-^ exchanger. Addition of Cl^-^ back to the external solution decreased the pH_i_ indicating HCO_3_^-^ efflux through the exchanger. Under these conditions, the initial rate of recovery from alkalosis reflects the activity of the Cl^-^/HCO_3_^-^ exchanger [(-*J*(B^-^/min)]. Using this technique, we showed that the rate of acidification significantly reduced in AQP1 KO (42 ± 3.2%) vs. WT ducts indicating that pancreatic ductal HCO_3_^-^ secretion is impaired in the absence of AQP1 (**Figures 2F,G**).

### AQP1 Expression Decreased in CFTR KO Mice

**FIGURE 3:**
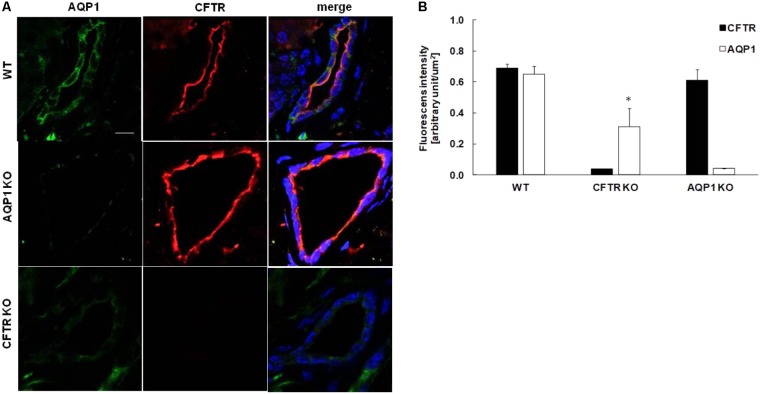
Expression of AQP1 and CFTR in the pancreas of knock out mice. **(A)** Representative immunofluorescence staining of AQP1 and CFTR in wild type (WT, upper line), AQP1 knock out (AQP1 KO, middle line) and CFTR KO (bottom line) mice. Pictures were taken at 40× magnification. Pancreas slices were excited at 405 (Dapi), 488 (Alexa fluor 488) and 568 (Alexa fluor 568) nm and emissions were collected at 453, 516, and 603 nm, respectively. Scale bar represents 10 μm. **(B)** Summary bar chart shows the mean fluorescence intensity in the ductal cells normalized to the ductal area and expressed in arbitrary units/μm^2^. Data are presented as means ± SEM. ^∗^*p* ≤ 0.05 vs. WT (AQP1), *n* = 5.

The decreased HCO_3_^-^ secretion in AQP1 KO mice indicates that beside the transport of water, AQP1 interacts with one or more ion transporters which are involved in HCO_3_^-^ secretion. The CFTR Cl^-^ channel plays essential role in ductal HCO_3_^-^ secretion by maintaining a luminal [Cl^-^] which is necessary for HCO_3_^-^ efflux through the Cl^-^/HCO_3_^-^ exchanger. Several studies presume a physical interaction between the CFTR Cl^-^ channel and certain AQP isoforms (Schreiber et al., 1999; Cheung et al., 2003; Jesus et al., 2014a,b). Colocalization of this two channel has also been found in the human pancreas (Burghardt et al., 2003). In the following step, we performed immunostaining on the pancreas of AQP1 and CFTR KO mice in order to characterize the possible relation between the two channels. In WT mice, AQP1 expression was detected throughout the whole plasma membrane, whereas expression of CFTR exclusively localized to the apical membrane of the ducts (**Figure 3A**). The absence of AQP1 caused a slight but not significant decrease in the expression of CFTR, indicating that the impaired HCO_3_^-^ secretion in the AQP1 KO mice is not due to the decreased expression of CFTR (**Figure 3A**, middle line). Interestingly we have found that expression of AQP1 dramatically decreased in the intra/interlobular ducts of CFTR KO mice, especially at the apical membrane (**Figure 3A**, bottom line and **Figure 3B**). We have also found that the absence of CFTR did not affect the expression of AQP1 in the blood vessels indicating that some kind of interaction may exist between these two channels in the pancreatic ductal cells. Although further investigations are needed to clarify whether the two channels are able to regulate each others function, expression or trafficking.

### Expression and Function of AQPs Significantly Decreased After Bile Acid Treatment

**FIGURE 4:**
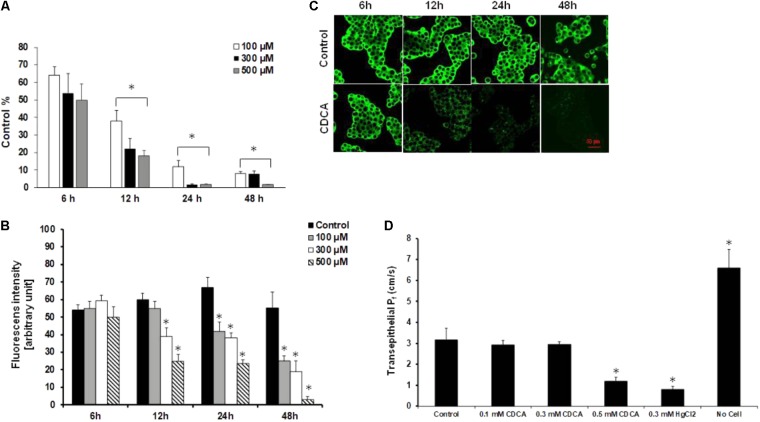
Effect of chenodeoxycholic acid on the expression and activity of AQP1. **(A)** Capan-1 cells were treated with chenodeoxycholic acid (CDCA) for 6, 12, 24, and 48 h and the relative gene expression of AQP1 was investigated by real-time PCR. Data represent mean ± SEM of three, independent experiments. **(B)** Protein expression of AQP1 was investigated by immunocytochemistry in Capan-1 cells. Protein expression was measured from the mean fluorescence intensity, normalized to the total cell number and expressed in arbitrary units. Data represent mean ± SEM of three, independent experiments. **(C)** Representative immunofluorescence staining of Capan-1 cells show the expression of AQP1 after the treatment with CDCA (500 μM) for 6, 12, 24, and 48 h. **(D)** Summary bar chart shows the changes in osmotic transcellular water movement (P_f_) of Capan-1 cells in the presence of various concentrations of CDCA. Transwell without cells was used for absolute positive control. Data represent mean ± SEM of three, independent experiments. ^∗^*p* ≤ 0.05 vs. Control.

In the next step, we studied the effect of pancreatitis-inducing factors on the expression of AQP1. Gallstone obstruction and heavy alcohol consumption are the two major causes of pancreatitis. Capan-1 cells were treated with CDCA and GCDCA (100, 300, and 500 μM) (Venglovecz et al., 2008, 2011; Muili et al., 2013), EtOH (1, 10, and 100 mM), POA and POAEE (10, 100, and 200 μM) (Criddle et al., 2006; Judak et al., 2014; Maleth et al., 2015) for 6, 12, 24, and 48 h and the mRNA expressions of AQP1 were analyzed by RT-PCR. Among the investigated agents, CDCA had the most marked effect, it dose- and time-dependently decreased the expression of AQP1 (**Figure 4A**). GCDCA, POA, and POAEE caused a significant decrease at 12 and 24 h, primarily at higher doses, which partially regenerated after 48 h in the continuous presence of the agents (**Supplementary Figures 1A–C**). In contrast to bile acids, EtOH initially (24 h) increased the expression of AQP1 that was followed by a decrease (**Supplementary Figure 1D**). In order to decide whether the downregulating effect of CDCA can also be observed at protein level, we performed immunostaining on the CDCA-treated cells, using specific antibodies against AQP1. CDCA dose- and time-dependently decreased the protein expression of AQP1 was consistent with the PCR data (**Figure 4B**). Representative ICC pictures show that incubation with 500 μM CDCA time-dependently decreased the AQP expression in the cells (**Figure 4C**). The effect of CDCA on the activity of AQPs was also investigated. As shown in **Figure 4D**, 100 and 300 μM CDCA had no effect on the transepithelial water movement, however, at 500 μM P_f_ was significantly impaired.

### Acute Pancreatitis Is Aggravated in AQP1 KO Mice

**FIGURE 5:**
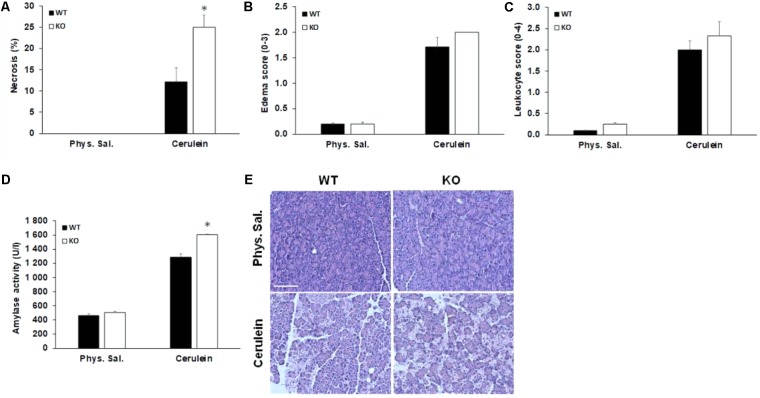
Characterization of acute pancreatitis in AQP1 knock out mice. **(A)** Pancreatic cell necrosis, **(B)** edema, **(C)** leukocyte infiltration, and **(D)** serum amylase activity (U/l) were measured in wild-type (WT) and AQP1 knock out (KO) mice after induction of pancreatitis. Control animals received the same amount of saline. Data are presented as means ± SEM. ^∗^*p* ≤ 0.05 vs. WT. **(E)** Representative histological images show pancreatic sections from control (physiological saline) and cerulein-treated animals. Phys sal., physiological saline. Scale bar represents 50 μm.

In order to test the hypothesis that AQP1 may be involved in the pathomechanism of pancreatitis, we investigated the severity of cerulein-induced AP in WT and AQP1 KO mice. WT and AQP1 KO mice were given 10 hourly injections of either physiological saline (control) or supramaximal doses of cerulein (50 μg/kg per injection) i.p. to induce AP. After saline injection, the pancreas had normal histology in both WT and KO animals. In contrast, i.p. injections of cerulein caused extensive cell damage both in the WT and KO animals. The extension of pancreatic necrosis were markedly higher in the AQP1 KO (25 ± 2.8%) vs. WT mice (12.1 ± 3.2%), whereas no differences were observed in the extent of edema and in the infiltration of inflammatory cells (**Figures 5A–C**). Serum amylase activities were significantly higher in KO (1605 ± 6 U/l) vs. WT mice (1285 ± 51 U/l) after the induction of AP (**Figure 5D**). As shown on the representative histological images (**Figure 5E**), the rate of pancreatic necrosis was more extensive in AQP1 KO mice. Overall, these results indicate that in the absence of AQP1 the course of pancreatitis is more severe.

### Expression of AQP1 Is Decreased in Acute and Chronic Pancreatitis

**FIGURE 6:**
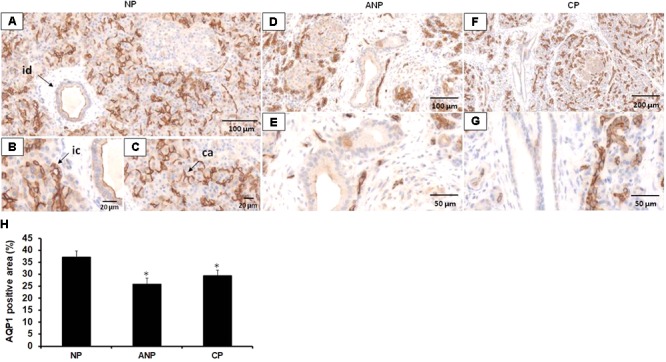
Expression of AQP1 in patients with acute and chronic pancreatitis. Representative histological images show expression and localization of AQP1 in normal human pancreas (NP, **A–C**) acute necrotizing pancreatitis (ANP, **D,E**) and chronic pancreatitis (CP, **F,G**) samples. AQP1 immunoreactivity was detected in inter/intralobular and intercalated ducts, in the acinar and centroacinar cells of the normal pancreas. In contrast, expression of AQP1 strongly decreased in ANP and CP. id, interlobular duct; ic, intercalated duct; ca, centroacinar cells. **(H)** Intensity of diaminobenzidine staining was measured in pancreas samples by ImageJ software and expressed as percentage of total pancreatic area. ^∗^*p* ≤ 0.05 vs. normal pancreas, *n* = 10–12.

Since we found that the lack of AQP1 exacerbates the course of cerulein-induced pancreatitis in mice, we tested whether this water channel is also involved in the pathomechanism of pancreatitis in humans. Therefore, in the next step we investigated the expression of AQP1 in pancreatic tissues samples obtained from five patients with ANP and five patients with CP. Control pancreatic tissue were obtained from tumor-free tissue surrounding neuroendocrine pancreatic tumors. In order to localize AQP1, IHC was performed. In the normal pancreas, strong AQP1 immunoreactivity was detected in the intra/interlobular and intercalated ducts, the acinar and centroacinar cells (**Figures 6A–C**). The staining in acinar cells and smaller ducts is mainly localized to the lateral and apical surface of the cells (**Figures 6B,C**). In the interlobular ducts, the apical plasma membrane was positive to AQP1 with some cytoplasmic staining, whereas Langerhans islets were completely negative for AQP1. These results are consistent with previous observations (Burghardt et al., 2003). In the ANP and CP pancreatic tissue sections the expression of AQP1 strongly reduced in the interlobular ducts, whereas intralobular and intercalated ducts still exhibited weak to moderate AQP1 immunoreactivity (**Figures 6D–G**). In case of acinar cells, the expression of AQP1 slightly decreased in the inflamed pancreas, especially in ANP. Quantification of DAB intensity showed that AQP1 staining was significantly higher in normal pancreas vs. the ANP or CP groups (**Figure 6H**).

## Discussion

Acute and chronic pancreatitis are serious disorders characterized by inflammation and injury of the gland. Gallstones and heavy alcohol consumption are responsible for approximately 80% of all AP patients (Parniczky et al., 2016), whereas alcohol abuse is the primary cause of CP (Szucs et al., 2017). Although the therapy improved a lot in the last few years the morbidity and mortality of pancreatitis is still excessively high (Parniczky et al., 2016). Defects in ductal fluid secretion have been proposed as an important factor in the pathomechanism of pancreatitis (Hegyi et al., 2011b). This fluid is high in HCO_3_^-^ which prevents the premature activation of zymogens and provides an optimal environment in the duodenum for the action of digestive enzymes. The secreted fluid is also beneficial from the point of view that washing out the potential toxic factors such as activated digestive enzymes or bile acids from the ductal tree and therefore prevents the pancreas from their damaging effects (Venglovecz et al., 2008). AQP1 is a water channel that extensively expressed in the acinar and ductal cells where mediates pancreatic fluid production. Several studies indicate that altered expression or localization of AQPs associates with different gastrointestinal disorders, such as gastritis or diarrhea; therefore, many studies have been conducted to identify the specific role of particular AQP isoforms. Although, AQP1 plays an essential role in pancreatic physiology its function under pathological conditions is not known.

In the present study, we showed that (i) bile acids strongly decrease the expression of AQPs in pancreatic ductal cells, (ii) chronic or acute inflammation of the pancreas is associated with decreased expression of AQP1, (iii) the absence of AQP1 reduces ductal fluid and HCO_3_^-^ secretion, (iv) and induces a more severe pancreatitis in a cerulein-induced pancreatitis model.

Basically there are two ways for water transport. One is the paracellular way along the osmotic gradient generated by the ion transporters and the other one is the transcellular pathway through the AQPs. In case of the pancreatic ducts, it is not clear which pathway is the dominant. The pancreatic ductal epithelium is a moderately leaky epithelium which favors the paracellular movement of water (Novak and Greger, 1988). Nevertheless, administration of luminal Hg^2+^, a non-specific inhibitor of AQPs, decreased ductal fluid secretion by more than 90% in rat pancreatic ducts, which strengthen the importance of transcellular pathway (Ko et al., 2002). In the present study, we demonstrated that pancreatic ductal cells express functionally active AQPs. We have identified the presence of AQP1, -3, and -5 on the ductal cells that are consistent with previous observations (Burghardt et al., 2003). Treatment of the cells with pancreatitis-inducing agents, mostly decreased the expression of AQPs both at mRNA and protein levels. Among the investigated agents, CDCA had the biggest effect, which dose- and time-dependently decreased the expression of AQP1. These data are partly consistent with previous findings in the colon, where bile acid treatment reduced the protein expression of AQP3, whereas increased the levels of AQP7 and -8 (Yde et al., 2016). In case of EtOH, the expression of AQPs initially increased (presumably due to a compensatory mechanism), then a decrease was observed, similarly to the rat stomach, where intragastrical administration of 1 mM EtOH caused analogous changes in the expression of AQPs (Bodis et al., 2001). High concentration of CDCA also decreased the activity of the water channels, which is somewhat related to our previous findings on guinea pig pancreatic ducts, where 1 mM CDCA strongly inhibited ductal HCO_3_^-^ and thus fluid secretion (Venglovecz et al., 2008). The involvement of AQP1 in epithelial water movements has been described in various tissues, although there is still no consensus among researchers regarding the functional importance of this water channel (Marples, 2000). In cholangiocytes, the absence of AQP1 does not affect the fluid secretion (Mennone et al., 2002), although inhibition of the secretin-induced translocation of AQP1 reduces bile flow more than half (Marinelli et al., 1997, 1999). It has been also demonstrated that inhibition of AQPs by HgCl_2_ dose-dependently reduced the osmotically induced volume increase in these cells (Roberts et al., 1994). In the kidney, the absence of AQP1 dramatically decreased the urine concentrating ability of mice due to the impaired water permeability of the proximal tubule, limb of Henle and vasa recta (Ma et al., 1998; Schnermann et al., 1998; Pallone et al., 2000). The importance of AQP1 has been also highlighted in the brain where cerebrospinal fluid production decreased by fivefold in mice lacking AQP1 (Oshio et al., 2004).

In order to determine the role of AQP1 in pancreatic fluid secretion, we used AQP1 KO mice. No differences were observed in body weight and physical appearance between WT and KO mice, although the lifespan of AQP1 deficient mice was slightly lower. Using both *in vitro* and *in vivo* approaches, we found that fluid and HCO_3_^-^ secretion significantly reduced in the absence of AQP1. These data are partly in contrast with previous observations demonstrating that defect in AQP1 expression cause only a small, but not significant decrease in the rate of stimulated pancreatic fluid secretion (Ma et al., 2001). Although this discrepancy can be explained by the different methods used for the measurement of pancreatic fluid. Nevertheless, other studies have found that decreased expression or function of AQP1 dramatically reduce ductal fluid secretion (Ko et al., 2002; Gabbi et al., 2008). Ko et al. (2002) have shown that luminal or basolateral administration of HgCl_2_ dose-dependently decrease the fluid secretory rate and osmotic water permeability of isolated pancreatic ducts. It has been also described that the defect in pancreatic fluid secretion in liver X receptor β-deficient mice is related to the decreased expression of AQP1 in the pancreatic ducts of these mice (Gabbi et al., 2008).

In order to identify the mechanism by which AQP1 influences ductal HCO_3_^-^ secretion, we investigated the relation of this channel with the CFTR Cl^-^ channel. CFTR is a cAMP-activated Cl^-^ channel, which primarily located at the apical membrane of epithelial cells and plays a crucial role in the maintenance of fluid homeostasis. Growing number of studies indicate that a molecular interaction exists between CFTR and certain AQP isoforms. It has been demonstrated that activation of CFTR by cAMP, increases the water permeability of the cells through the activation of AQP3 (Schreiber et al., 1999; Jourdain et al., 2014). The relation between AQPs and CFTR has been also confirmed in rat epididymis, where CFTR potentiates AQP9-mediated water permeability (Cheung et al., 2003). Moreover, a direct interaction between AQPs and CFTR has been also observed in Sertoli cells (Jesus et al., 2014a,b). Using double immunostaining we showed for the first time that AQP1 and CFTR are co-localized at the apical membrane of pancreatic ductal cells. Lack of CFTR significantly decreased the expression of AQP1, indicating that CFTR somehow controls the water permeability of ductal cells. Similar results have been found in respiratory epithelial cells, where in the presence of CFTR inhibitor or mutant CFTR the water permeability significantly decreased (Schreiber et al., 1999, 2000; Jourdain et al., 2014) which highlights the significance of this water channel in cystic fibrosis. In contrast, expression of CFTR does not depend on the presence of AQP1, since in the absence of this water channel, strong CFTR staining was detected. This result indicates that the decreased HCO_3_^-^ secretion in the AQP1 KO mice is not due to the impaired expression of CFTR. Nevertheless, we cannot exclude that although the expression of CFTR did not change but the channel does not work correctly, however, further functional experiments are needed to confirm this hypothesis.

There is more and more evidence that impaired pancreatic fluid secretion plays role in the pathomechanism of pancreatitis (Hegyi and Rakonczay, 2010; Pallagi et al., 2014; Maleth et al., 2015). So in the next step, we investigated whether the lack of AQP1 has any effects on the progression of pancreatitis. The loss of AQP1 itself does not damage the pancreas and does not cause pancreatitis in mice, which assuming the compensating effect of AQP5; however, induces a more severe disease progression. The involvement of AQP1 in the pathophysiology of pancreatitis has been already raised previously (Kitami et al., 2007). Kitami et al. (2007) have found that AQP1 expression decreased both in the ductal and acinar cells in a cerulein-induced pancreatitis model, which indicate that reduced levels of AQP1 may contribute to exocrine insufficiency. These findings are in accordance with our observation that expression of AQP1 decreased in the ductal and acinar cells of patients with AP or CP. The importance of AQP1 in the exocrine pancreas has been also confirmed by the fact that this water channel is abundantly expressed in the zymogen granules of acinar cells and plays an essential role in zymogen swelling and probably secretion (Cho et al., 2002). AQP12 is also expressed in the zymogen granule of acinar cells and huge amount of this isoform is present in the rough endoplasmic reticulum (Ohta et al., 2009). In the absence of AQP12, high concentration of cholecystokinin octapeptide makes the pancreas more susceptible to pancreatitis, presumably by the aberrant exocytosis of zymogen granules in these mice (Ohta et al., 2009). All of these previous observations indicate that AQP1 plays essential role both in ductal and acinar functions and we speculate that the absence of AQP1 makes the pancreas more sensitive in two ways: (1) ductal fluid secretion is not sufficient and (2) exocytosis of zymogen granules is impaired.

In this study, we provided an overview regarding the expression and role of AQP1 in the physiology and pathophysiology of the pancreas. Our data indicate that AQP1 interacts with the CFTR Cl^-^ channel and takes part in the formation of pancreatic fluid. Moreover, we have found that AQP1 plays role in the pathology of pancreatitis. We hypothesize that absence of the channel makes the pancreas more sensitive to pancreatitis, probably due to the decreased pancreatic fluid and HCO_3_^-^ secretion. Our novel findings not only help to understand the pathomechanism of pancreatitis better, but open up new therapeutic opportunities in the treatment of the disease.

## Author Contributions

PP performed microfluorimetric and videomicroscopy experiments. LK, ÁZ, and LP were involved in molecular biology experiments. AB, MS, and J-PK performed MRI and JM and ML interpreted the MRI pictures. Genotyping and breeding of AQP mice were done by EB and EG. Pancreatitis was induced by ZB. KB and ET did the immunostainings on the human and mice pancreatic samples, respectively, and quantified fluorescence intensity. ZR was involved in data interpretation and edited the manuscript. VV was involved in all of the above mentioned experiments, analyzed the data, and drafted the manuscript. PH supervised the project and edited the manuscript. All authors approved the final version of the manuscript.

## Conflict of Interest Statement

The authors declare that the research was conducted in the absence of any commercial or financial relationships that could be construed as a potential conflict of interest.

## Supplementary Material

FIGURE S1 Expression of AQP after various treatments. Capan-1 cells were treated with **(A)** glycochenodeoxycholic acid (GCDCA), **(B)** palmitoleic acid (POA), **(C)** palmitoleic acid ethyl ester and **(D)** ethanol (EtOH) for 6, 12, 24, and 48 h and the relative gene expression of AQP1 was investigated by real-time PCR. Data represent mean ± SEM of three, independent experiments. ^∗^*p* ≤ 0.05 vs. Control.
