# Supplementary figures and images for: The Importance of Aquaporin 1 in Pancreatitis and Its Relation to the CFTR Cl- Channel
Source: Front Physiol. 2018 Jul 12;9:854. doi: 10.3389/fphys.2018.00854 (PMC6052342; doi:10.3389/fphys.2018.00854)

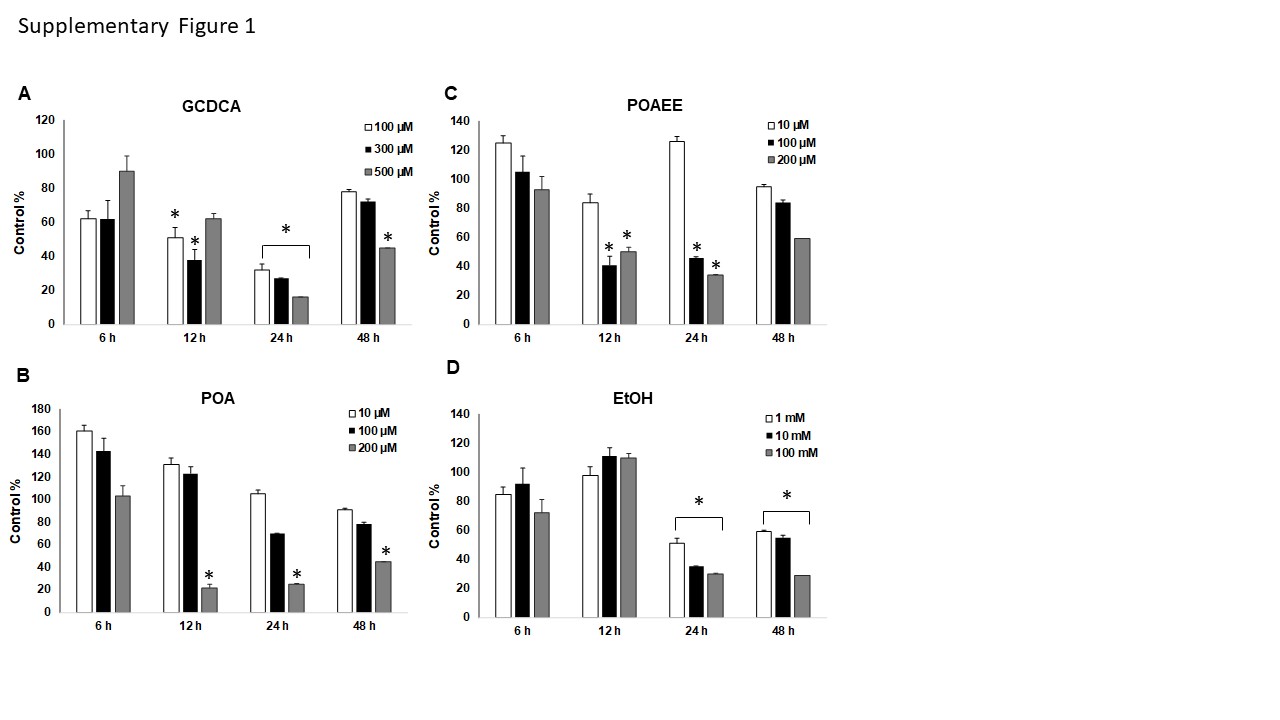

Supplement: FIGURE S1 — Expression of AQP after various treatments. Capan-1 cells were treated with (A) glycochenodeoxycholic acid (GCDCA), (B) palmitoleic acid (POA), (C) palmitoleic acid ethyl ester and (D) ethanol (EtOH) for 6, 12, 24, and 48 h and the relative gene expression of AQP1 was investigated by real-time PCR. Data represent mean ± SEM of three, independent experiments. ∗p ≤ 0.05 vs. Control. [file Image_1.JPEG]
